# Type I collagen hydrogels as a delivery matrix for royal jelly derived extracellular vesicles

**DOI:** 10.1080/10717544.2020.1818880

**Published:** 2020-09-14

**Authors:** Orlando J. Ramírez, Simón Alvarez, Pamina Contreras-Kallens, Nelson P. Barrera, Sebastian Aguayo, Christina M. A. P. Schuh

**Affiliations:** a Facultad de Medicina, Centro de Medicina Regenerativa, Clínica Alemana Universidad del Desarrollo, Santiago, Chile; b Faculty of Biological Sciences, Department of Physiology, Pontificia Universidad Católica de Chile, Santiago, Chile; c Faculty of Medicine, Dentistry School, Pontificia Universidad Católica de Chile, Santiago, Chile; d Institute for Biological and Medical Engineering, Schools of Engineering, Medicine and Biological Sciences, Pontificia Universidad Católica de Chile, Santiago, Chile

**Keywords:** Wound healing, *Apis mellifera*, regenerative medicine, drug delivery, extracellular vesicle delivery

## Abstract

Throughout the last decade, extracellular vesicles (EVs) have become increasingly popular in several areas of regenerative medicine. Recently, *Apis mellifera* royal jelly EVs (RJ EVs) were shown to display favorable wound healing properties such as stimulation of mesenchymal stem cell migration and inhibition of staphylococcal biofilms. However, the sustained and effective local delivery of EVs in non-systemic approaches – such as patches for chronic cutaneous wounds – remains an important challenge for the development of novel EV-based wound healing therapies. Therefore, the present study aimed to assess the suitability of type I collagen -a well-established biomaterial for wound healing – as a continuous delivery matrix. RJ EVs were integrated into collagen gels at different concentrations, where gels containing 2 mg/ml collagen were found to display the most stable release kinetics. Functionality of released RJ EVs was confirmed by assessing fibroblast EV uptake and migration in a wound healing assay. We could demonstrate reliable EV uptake into fibroblasts with a sustained pro-migratory effect for up to 7 d. Integrating fibroblasts into the RJ EV-containing collagen gel increased the contractile capacity of these cells, confirming availability of RJ EVs to fibroblasts within the collagen gel. Furthermore, EVs released from collagen gels were found to inhibit *Staphylococcus aureus* ATCC 29213 biofilm formation. Overall, our results suggest that type I collagen could be utilized as a reliable, reproducible release system to deliver functional RJ EVs for wound healing therapies.

## Introduction

Exosomes and extracellular vesicles (EVs) have become one of the upcoming topics in regenerative medicine. Discovered only in 1981 and hypothesized to be a cellular waste disposal system, they are now known as an integral part of cellular communication (Trams et al., 1981; Pan & Johnstone, 1983; Bang & Thum, 2012). In the last decade, the potential of EVs as a therapeutic option has gained considerable interest for various conditions, including cancer, chronic wounds, liver regeneration, and ischemic pathologies, among others (Li et al., 2013; Doeppner et al., 2015; Teng et al., 2015; Wang et al., 2015; Zhang et al., 2015; Ha et al., 2016; Yang & Wu, 2018). The most commonly used route of delivery is systemic, intravenous injection of EVs in solution. While systemic delivery displays a number of advantages such as rapid, easy, and standardizable delivery, the disadvantages cannot be ignored. Among these are (a) high costs of production to obtain large quantities of EVs for systemic delivery in humans (Colao et al., 2018); (b) poor targeting to the site of interest; (c) rapid recognition by the immune system leading to EV degradation in the liver and spleen (Yi et al., 2020); and (d) non-suitability of an intravenous approach for tissues or conditions with reduced vascular support, e.g. cartilage tissue or chronic wound sites (Sophia Fox et al., 2009; Frykberg & Banks, 2015).

Most notoriously, chronic wounds are known to react poorly to systemic treatments, making management options especially difficult. They display two mayor challenges: wound healing impairment due to dysregulation in the cellular response, and propensity to infections as a result of an altered immune response amid decreased vascular support supplying the wound bed with immune cells (Frykberg & Banks, 2015). Current clinical management consists of wound bed debridement and subsequent wound coverage with antibacterial dressings, hydrogels or negative pressure therapy, among others (Fleck & Simman, 2010; Han & Ceilley, 2017). Cell therapy approaches have mostly been focusing on mesenchymal stem cells due to their pro-angiogenic and anti-inflammatory effects, but issues such as cell homing and poor viability over time remain as important concerns (Wagner et al., 2009; Lee et al., 2016). Thus, EVs have emerged as an interesting alternative in order to circumvent these cell-therapy limitations. However, only a limited number of studies have been performed using EVs in a local delivery system, using chitosan or hyaluronic acid based scaffolds with different MSC-derived EVs (Tao et al., 2017; Wang et al., 2019).

As a novel source of active exosomes, we have recently reported the presence of EVs in honeybee *Apis mellifera* royal jelly (Schuh et al., 2019). Royal jelly has demonstrated antimicrobial and pro-regenerative characteristics in the past in several wound-associated conditions, and pre-clinical studies employing this substance described an improvement in a number of conditions including mucositis or infected ulcers (Watanabe et al., 2013; Siavash et al., 2015). Isolated royal jelly extracellular vesicles (RJ EVs) displayed strong antibacterial and biofilm-inhibiting properties and stimulated migration in mesenchymal stem cells-characteristics favorable for chronic wound treatments (Schuh et al., 2019). However, an ideal local delivery matrix for RJ EVs has not yet been developed. One of the most widely used biomaterials in wound healing is type I collagen, which has demonstrated excellent results by stimulating wound fibroblasts to deposit and organize collagen and recruiting wound-associated immune cells, while keeping a moist environment (Fleck & Simman, 2010). The structure of type I collagen gels appears to be favorable for EV encapsulation, given it is a natural polymer comprised of a triple helix configuration with the ability of forming fibrils that are cross-linked into a 3D porous mesh (Antoine et al., 2014). Therefore, in this present study we assessed the suitability of type I collagen gels as a delivery system for RJ EVs. We evaluated release kinetics regarding their suitability to serve in a prolonged wound healing environment, ultra-structural changes of the material in presence of RJ EVs, as well as functionality of the RJ EVs after release. Our results suggest that type I collagen can be utilized as a reliable, reproducible release system to deliver functional RJ EVs.

## Materials and methods

### Exosome isolation and characterization

Exosome isolation was based on a previously published protocol (Schuh et al., 2019). Briefly, royal jelly (Apícola del Alba, Chile) was diluted in particle-free phosphate buffered saline 1:40 (pf-PBS). Subsequently, samples were centrifuged at 500 × *g*, 1000 × *g*, 1500 × *g*, and 2000 × *g* for 15 min each and filtered (0.2 µm polystyrene filter). Resulting supernatant was ultra-centrifuged at 100.000 × *g* for 60 min (Hanil 5 fixed rotor ultracentrifuge, Hanil, Korea). The pellet containing exosomes was resuspended in pf-PBS and stored at −80 °C until further use.

For nanoparticle tracking analysis (NTA), samples were thawed shortly before measurement, vortexed and diluted 1:100 with pf-PBS. Subsequently, samples were injected manually and measured at camera level 8 in temperature-controlled environment (25 °C) for 60 s per sample (NanoSight NS 3000, Malvern, UK).

### Type I collagen gel preparation

To determine a collagen gel concentration favorable for exosome release, stock solutions of 3 mg/ml, 2 mg/ml, and 1 mg/ml in hydrochloric acid (Gibco, US) were mixed with 10× MEM (Gibco, US) in a ratio of 8:1. After neutralization with 1 M Sodium Hydroxide, 1-part microvesicles was added at a concentration of 2.5 × 10^9^/ml to complete 10-parts solution. Control gels not carrying microvesicles were adjusted with 1 part pf-PBS. One hundred microliter gels were left to polymerize at 37 °C in a 96-well plate and subsequently covered with 100 µl PBS.

### Microvesicle release from collagen gels

Gels were incubated with 100 µl pf-PBS and samples for NTA analysis were taken at day 1, 3 and 7, and stored at −80 °C. For NTA analysis, samples were diluted 1:10 with pf-PBS and measured as described above. RJ EV samples were normalized on respective control to eliminate unspecific background (e.g. collagen debris or aggregates of PBS; Figure 2(C)).

### Atomic force microscopy of collagen substrates

Collagen gels at a concentration of 2 mg/ml (favorable concentration determined in microvesicle release experiments) were prepared on ice, with and without RJ exosomes (2.5 × 10^9^/ml). Fifty microliter gels in a 96 well plate were left to polymerize for 30 min at 37 °C. Gels were then covered with PBS for 5 h and mildly fixed by immersion in paraformaldehyde 4% (Sigma, US) for 30 mins. Samples were immediately washed twice with distilled water and kept at 4 °C until atomic force microscopy (AFM) experiments (within 24 h). For AFM imaging, gels were physiosorbed onto a clean glass cover slips and immediately placed under a MFP 3D-SA AFM (Asylum Research, US). Images of 5 × 5 µm and 2 × 2 µm were obtained for both conditions in AC mode, employing TAP300GD-G cantilevers (*k* ∼ 40 N/m, ∼300 kHz, BudgetSensors, Bulgaria). Height and amplitude channel data was recorded and processed with proprietary Asylum Research AFM software (v16.10.211).

### Biological activity of RJ EVs released from collagen gels

For all cell culture assays, cells were cultivated in Dulbecco’s Modified Eagle Medium (Gibco US) supplemented with 10% fetal bovine serum (FBS; Gibco, US), 2 mM l-Glutamine, 1% Penicillin/Streptomycin (both Sigma, US). Cells were kept in a humidified incubator at 37 °C and 5% CO_2_.

### Uptake of RJ EVs into fibroblasts

Release patterns found with NTA analyses were verified using an EV uptake assay. Cells were analyzed on days 1, 3, and 7. For each analysis, 3T3-L1 cells were seeded at a concentration of 1.25 × 10^4^ cells/cm^2^ and left to adhere overnight to ensure equal confluence. Microvesicles were stained with CFSE membrane dye (Carboxyfluorescein succinimidyl ester; Thermo Fisher, US) as described previously (Schuh et al., 2019), and incorporated into 2 mg/ml collagen gels at a concentration of 2.5 × 10^9^/ml. Gels containing CFSE-stained RJ EVs were co-localized with the cells using a Transwell insert (6.5 mm diameter, 8 µm polycarbonate membrane; Corning, US). CFSE-stained RJ EVs added directly into the media served as positive control. After overnight incubation, cells were washed 3× with pf-PBS to remove residual RJ EVs and fixed with 4% formaldehyde for 30 min. Nuclei were stained with Hoechst (Cell Signaling, US) and samples were washed 3× with PBS prior to mounting. Images were taken on an Olympus Fluoview 10I microscope.

### Cellular migration assay

Biological activity of RJ EVs released from collagen gels was evaluated in a scratch assay on day 1, 3, and 7 after gel preparation. Human dermal neonatal fibroblasts (HdnF; Thermo Fisher, US) were seeded in a 24-well plate (5 × 10^4^/cm^2^) and left to form a confluent cell layer for 24 h. Collagen gels with and without RJ EVs were co-localized with the seeded cells using a Transwell system over night. To initiate the assay, a scratch was inflicted (time point 0 h) using a pipette tip and the cell layer was washed with PBS to remove residual non-attached cells as well as FBS. Cells were kept in medium without FBS and Transwells were added for continuous exposure to EVs. Scratch closure was assessed 4, 8, 12, and 24 h after scratch infliction and compared to time point 0 h.

### BrdU proliferation assay

Proliferation of HdnFs was assessed on day 1, 3, and 7 after gel preparation. Therefore, cells were seeded in a 24 well plate at a density of 2.5 × 10^4^/cm^2^ and left to attach for 24 h. Subsequently, 5-bromo-2′-deoxyuridine (BrdU colorimetric cell proliferation kit, Roche, US) was added to the wells at a final concentration of 100 µM and collagen gels with and without RJ EVs were co-localized with the cells using a Transwell system over night. Culture plates were fixed with FixDenat solution and incubated with anti-BrdU peroxidase (POD) antibody solution for 90 min at room temperature. Unbound antibody was removed by thorough washing with PBS (3 × 5 min) and wells were incubated with substrate solution (tetramethylbenzidine) for 15 min. The reaction was stopped with 1 mM H_2_SO_4_, and absorption was measured at 450 nm on a microplate reader (Tecan Sunrise, Austria).

### Collagen contraction assay

Interaction between collagen gels with and without RJ EVs and HdnFs was evaluated in a collagen contraction assay. Collagen gels were prepared as described above and 1.5 × 10^6^ cells per ml were added after neutralization and addition of RJ EVs. Collagen/RJ EV/HdnF mixtures were pipetted in 100 µl aliquots into 96-well plates and immediately placed at 37 °C in a humidified incubator for 60 min to allow polymerization and cell attachment. Subsequently, gels were detached from the sides of the well using a sterile needle and covered with 100 µl media containing 5% FBS. Gels without RJ EVs incubated in media containing 0%, 5%, and 20% FBS served as controls. To discriminate between effects of the RJ EVs on the contraction of the gel due to nano-structural changes, or due to effects on the cells, another control was added: HdnF were pre-conditioned with RJ EVs for 24 h (100 RJ EVs per seeded cell).

For analysis of gel contraction, media was removed after 24 h and gels were imaged using a standard laboratory camera. Gel size was analyzed using ImageJ software and images were normalized on well size.

### Biofilm inhibition assay

Inhibition of biofilm formation was assessed using the wound-relevant and known biofilm-forming strain *Staphylococcus aureus* ATCC 29213 (Bowler et al., 2001). *S. aureus* ATCC 29213 were maintained on Tryptic Soy Broth (TSB) agar plates (BD, USA), and grown for 24 h at 37 °C in aerobic conditions. Bacteria were prepared according to guidelines from the Clinical and Laboratory Standards Institute, CLSI (CLSI, 2014). For quantitative evaluation of biofilm formation, bacteria were grown in TSB with 1% glucose (TSBG) in 24-well plates. CFU was adjusted to 0.5 McFarland and subsequently diluted to 5 × 10^5^ CFU/ml, of which 400 µl were utilized per well (total: 2 × 10^5^). Transwells containing collagen with and without EVs were co-localized with the bacteria, Ampicillin (300 mM, 40 µl) served as positive control, and *S. aureus* in TSBG served as growth control. For quantitative analysis, biofilms were dried and stained with a crystal violet solution (CV, 0.1% in H_2_O; Sigma, US) for 15 min. Subsequently, plates were washed with H_2_O to remove excess stain and incubated with 95% ethanol to liberate the CV from the biofilm. Supernatants were transferred into a fresh 96-well plate, and biomass was determined by absorption at 590 nm (Tecan Sunrise, Tecan, Switzerland).

### Statistics

All data in this study are shown as mean ± standard deviation (SD). Statistical analysis was performed – depending on groups analyzed – using student *t*-test, one-way analysis of variance (ANOVA) or two-way ANOVA followed by Tukey’s range test for significant differences between the means. Significance was considered at *p* < .05. For statistical calculations, GraphPad Prism 5 for Mac OS X, Version 5.0 b (GraphPad Software, Inc., USA) was used.

## Results

### Characterization of EVs

**Figure 1.**
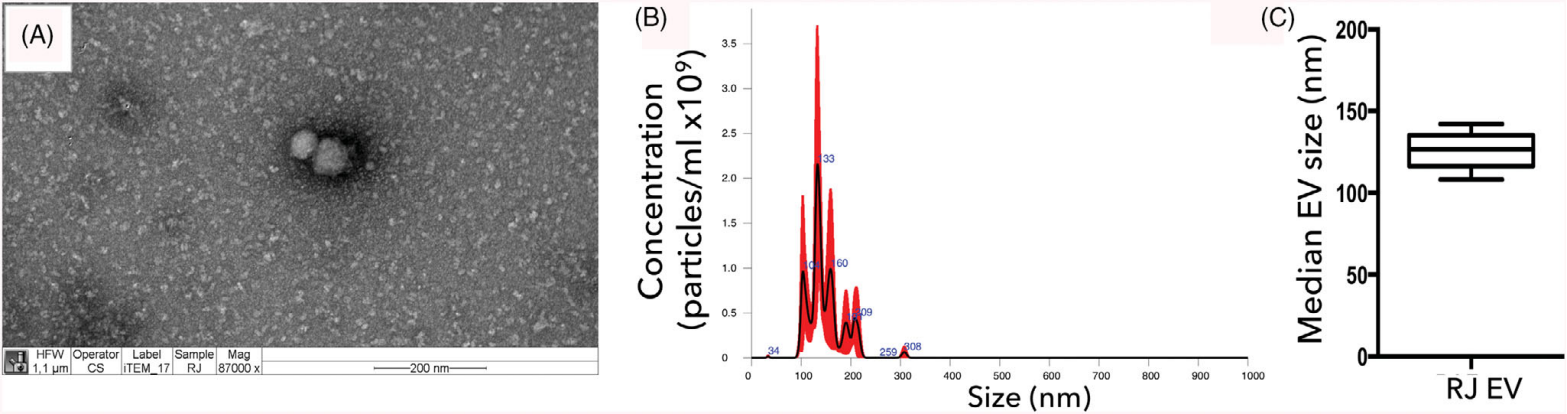
Characterization of RJ EVs; (A) Transmission electron micrograph of RJ EVs; scale bar = 200 nm; (B) representative NTA histogram of particle distribution; (C) NTA analysis of median particle size in nm; mean ± SD; *n* = 5.

The presence and size distribution of nanoparticles isolated from royal jelly using ultracentrifugation was assessed with NTA and TEM. All EV batches utilized for this study were verified to contain vesicles displaying the majority of their particles within the size range of exosomes (<150 nm; Figure 1(A)), with a mean median size of 122.6 nm (±9.8 nm; Figure 1(B,C)).

### Collagen concentration determines RJ EV release patterns

**Figure 2.**
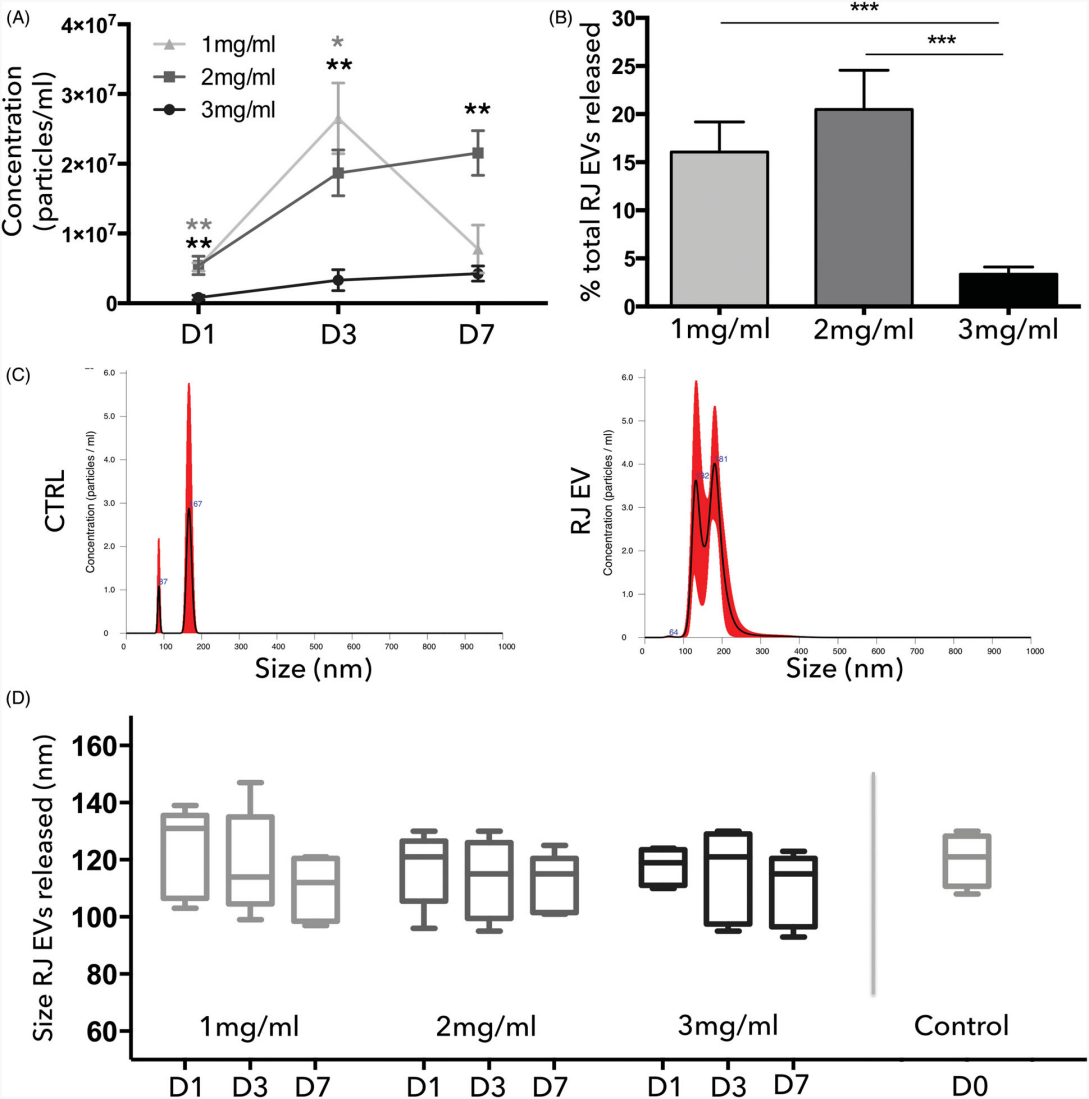
Release kinetics of type I collagen and RJ EVs; (A) NTA analysis of particles released into the surrounding medium from 1 mg/ml (light gray), 2 mg/ml (dark gray), and 3 mg/ml (black) collagen gels on day 1, 3, and 7; Concentrations displayed as particles/ml; (B) Cumulative RJ EVs released from 1 mg/ml, 2 mg/ml, and 3 mg/ml collagen gels after 7 d; data displayed as percentage of initial RJ EVs integrated into the gels; (C) representative NTA histogram of Ctrl (PBS incubated with collagen without RJ EVs) and RJ EVs released from collagen gels; (D) Median size of RJ EVs released from 1 mg/ml, 2 mg/ml, and 3 mg/ml collagen gels on day 1, 3, and 7 and prior to integration into collagen gels (control); A, B, D: *n* = 5; mean ± SD; statistics described in methods.

To determine the optimal collagen concentration for vesicle release over time, EV content in the surrounding media was assessed with NTA on day 1, 3, and 7 (representative NTA histogram of collagen control and RJ EVs shown in Figure 2(C)). It was observed that release patterns differed between studied groups and depended strongly on the collagen concentration used (Figure 2(A)). The highest collagen concentration (3 mg/ml) displayed a continuously low release pattern with no significant increase between day 1, 3, and 7, starting with 8.1 × 10^4^ particles/100 µl released on day 1, followed by 3.3 × 10^5^ on day 3 and 4.2 × 10^5^ on day 7. The total amount of particles released within 7 d (8.6 × 10^6^) was around 3.35% of the particle amount introduced into the gel (2.5 × 10^8^). The second collagen concentration tested, 2 mg/ml, displayed a continuous increase in EV release, with a significant increase between day 1 and day 3 (5.4 × 10^5^ and 1.8 × 10^6^, respectively), and a minor increase on day 7 (2.1 × 10^6^). In total, around 18.2% of EVs were released from the gel within 7 d. An interesting release pattern was observed in the 1 mg/ml group: starting on day 1 with approximately the same amount as 2 mg/ml (5.2 × 10^5^), followed by a significant increase (2.6 × 10^6^), and again a significant decrease to 7.7 × 10^5^, with a total 15% of EVs released (Figure 2(A,B)). Furthermore, NTA analysis revealed no significant change in RJ EV size between the groups and over time (Figure 2(D)).

### Released EVs are biologically active

#### AFM visualization of collagen gels releasing EVs

**Figure 3.**
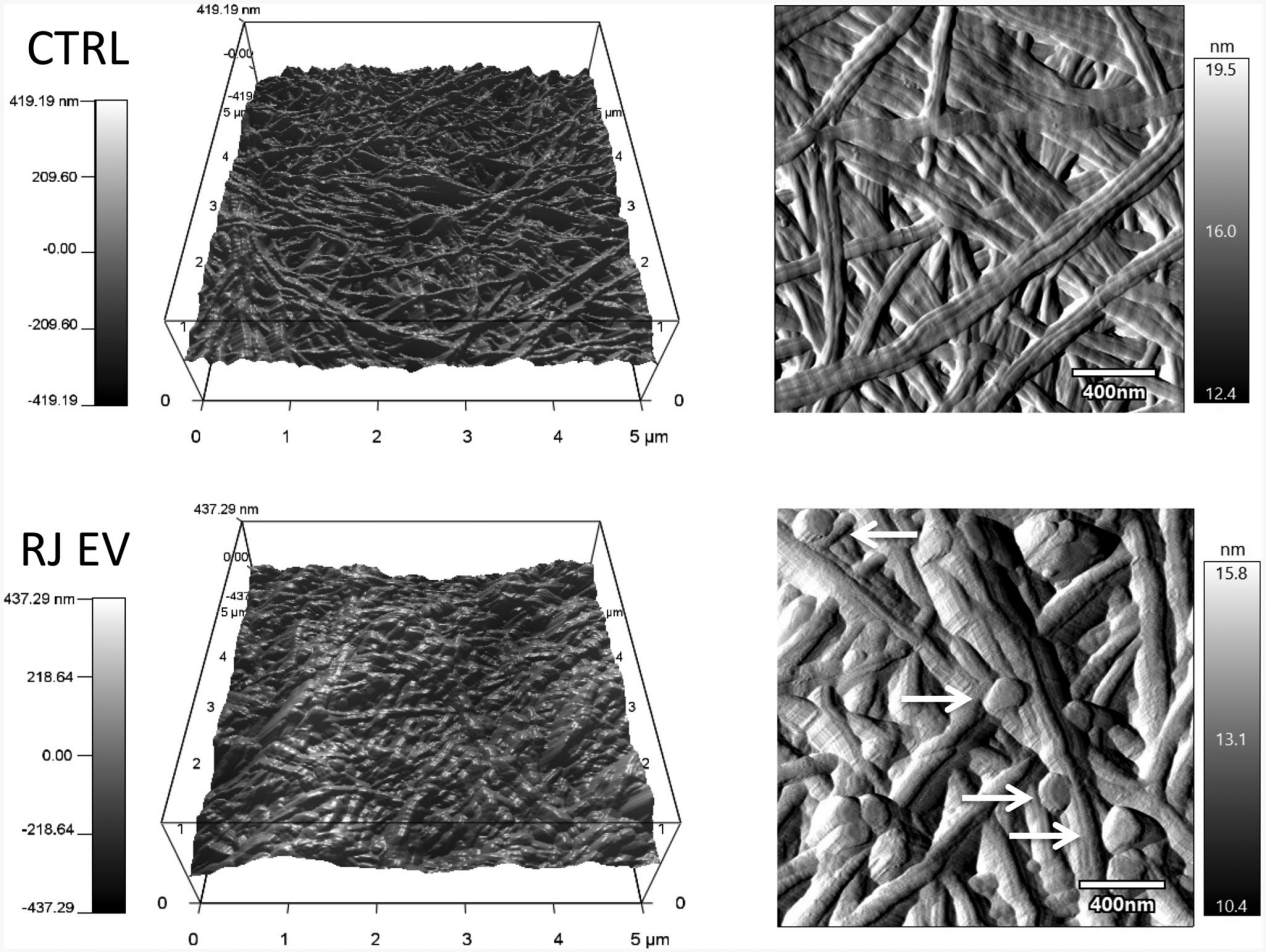
AFM imaging of 2 mg/ml collagen gels with and without RJ EVs; 3D height (5 × 5 μm) and amplitude (2 × 2 μm; scale bar = 400 nm) images of control collagen and collagen loaded with (2.5 × 10^9^/ml) RJ EVs, imaged in AC mode; Arrows indicate RJ EVs released from the collagen gel.

Imaging nanoparticles such as EVs is known to be complex and prone to pitfalls, especially when integrated into a biological matrix material. To obtain a relatively unaltered snapshot of RJ EV-collagen interaction, nondestructive AFM imaging was chosen. Mild fixation and physisorption allowed RJ EVs on both the surface and between collagen fibers to be imaged (Figure 3). RJ EVs were visualized as round-shaped and slightly flattened, displaying the typical EV size of around 100 to 200 nm. Qualitatively, the overall appearance of collagen gels loaded with RJ EVs was different compared to control collagen gels, including D-banding visualization and fiber arrangement (Figure 3).

#### Fibroblast internalize RJ EVs released from collagen gels

**Figure 4.**
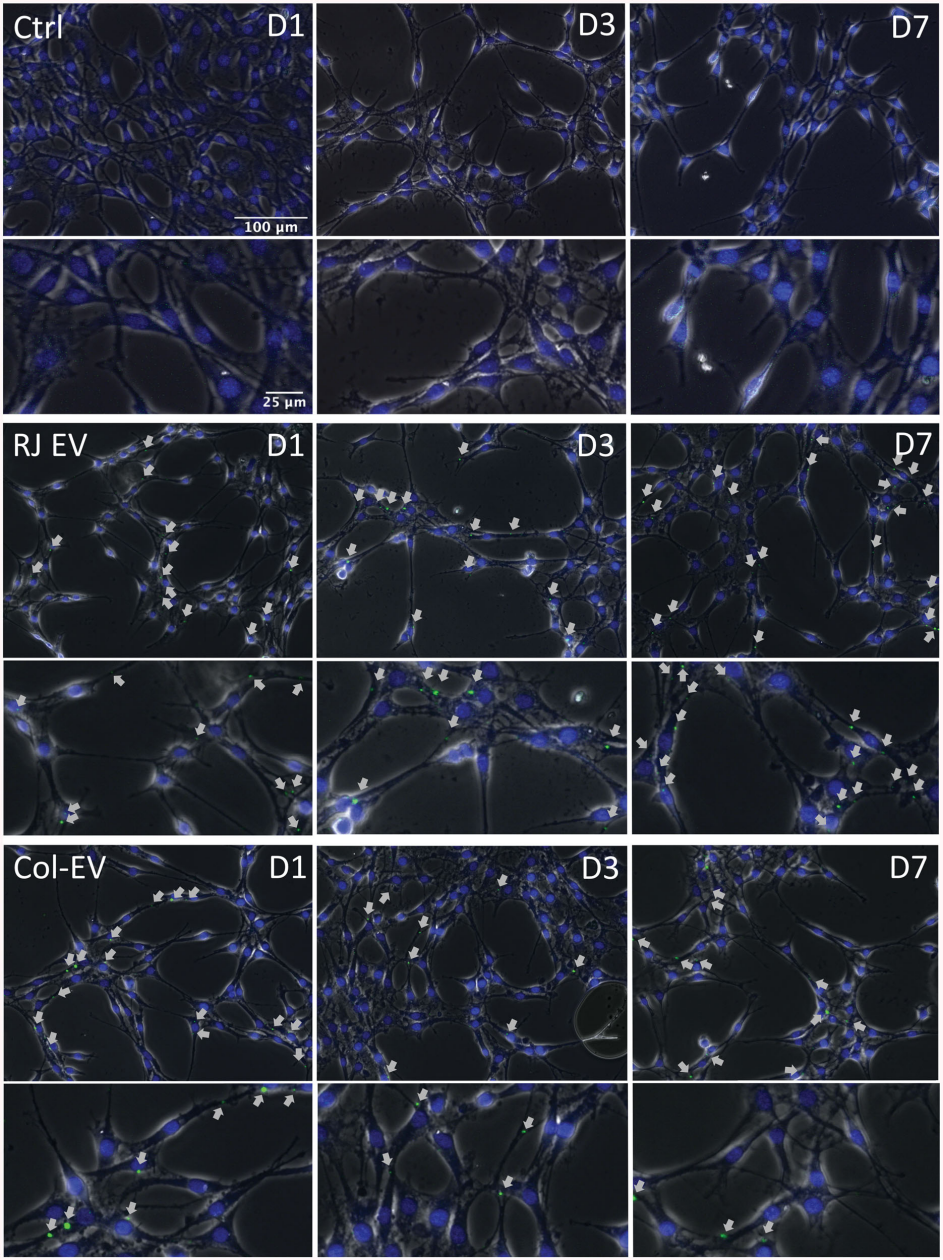
Integration of RJ EVs into 3T3 L1 cells; Confocal imaging of 3T3 L1 cells on day 1, 3, and 7 after colocalization with Transwells containing collagen gels with CFSE-ELVs (Col-EV); RJ EV displays control cells receiving CFSE RJ EVs 24 h before analysis; ctrl was not incubated with RJ EVs; nuclei stained with Hoechst; Arrows indicate EVs within cells; scale bar = 100 μm (upper panels per group) and 25 µm (lower panels per group).

In a first attempt to assess biological activity of RJ EVs released from collagen matrixes, their uptake into 3T3 L1 fibroblasts was assessed over time and compared to the uptake of RJ EVs freely available in the culture medium. Uptake into fibroblasts was observed on day 1, 3, and 7- with no apparent differences between the time points, as seen in Figure 4.

#### Increased migration and decreased proliferation of fibroblasts exposed to RJ EVs

**Figure 5.**
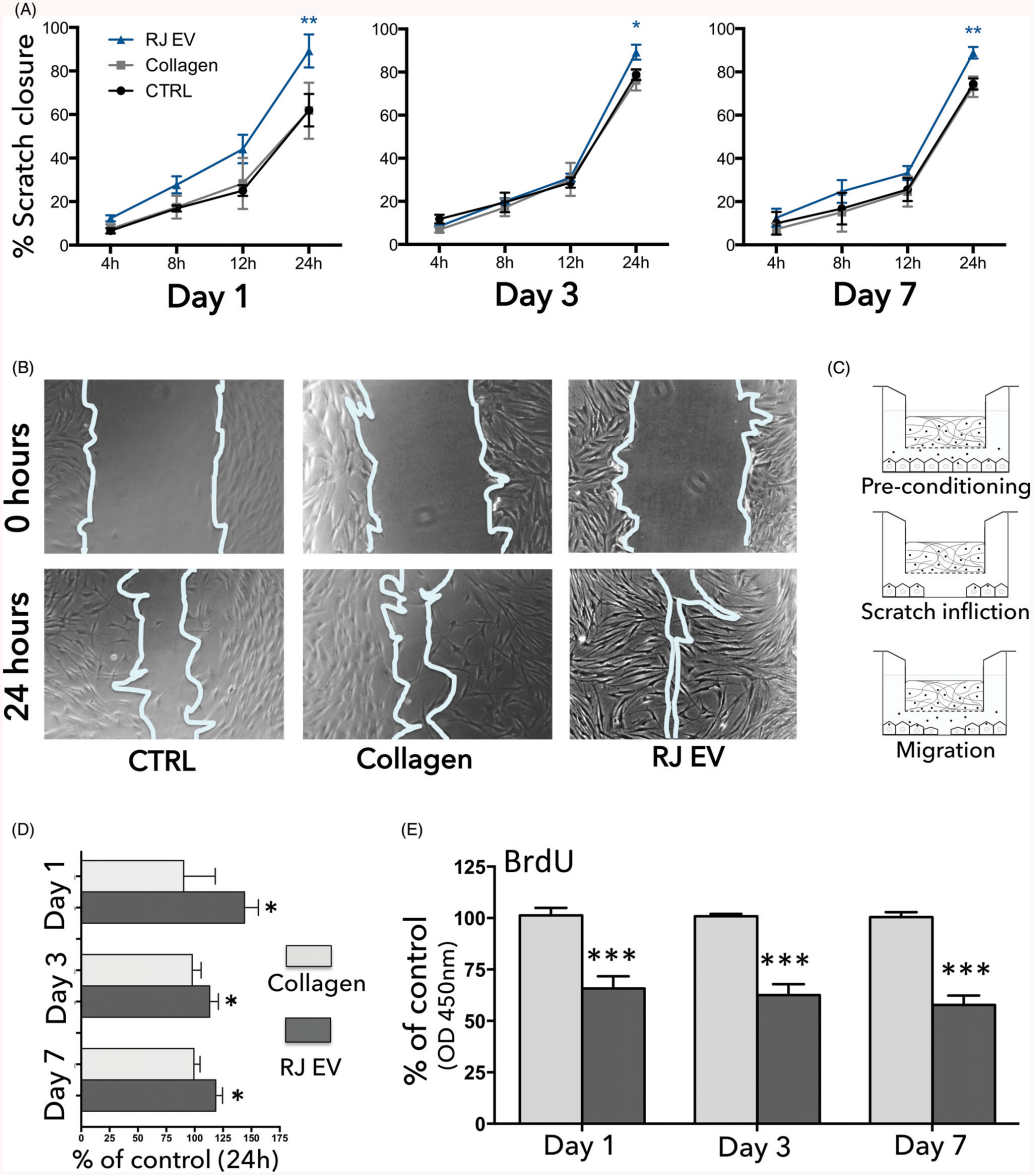
RJ EVs released from collagen gels promote HdnF migration and decrease proliferation; (A) Quantitative analysis of scratch closure (4, 8, 12, and 24 h); fibroblasts colocalized with collagen gels with and without RJ EVs compared to ctrl; data displayed as percentage scratch closure compared to 0 h; (B) Representative phase contrast micrographs at time point 0 h and 24 h; (C) Schematic representation of the experimental setup: fibroblasts were preconditioned for 24 h with RJ EVs released from collagen gels, subsequently a scratch was inflicted and cellular migration was assessed after 4, 8, 12, and 24 h; (D) Scratch closure of collagen gels with (dark gray) and without (light gray) RJ EVs after 24 h, as percentage of migration ctrl; (E) proliferation of HdnF evaluated with BrdU assay in presence of collagen gels with (dark gray) and without RJ EVs (light gray), measured at 450 nm; data displayed as percentage of control; A, C: *n* = 4; E: *n* = 5; mean ± SD; statistics described in methods.

After confirmation of prolonged stability and uptake into HdnFs over the time course of 7 d, the pro-migratory effect was assessed as well on day 1, 3, and 7 (representative images at 0 h and 24 h timepoints shown in Figure 5(B)). Collagen gels without RJ EVs in Transwells displayed no significant effect on cell migration and proliferation, and followed the migratory and proliferative behavior of cells in the control group (Figure 5(A,D,E)). Collagen gels releasing RJ EVs, however, considerably increased cellular migration (Figure 5(A,D)) as well as decreased proliferation (Figure 5(E)). This was reflected in significantly increased scratch closure on all time points assessed (days 1, 3, and 7), shown in Figure 5(B,D). A trend toward faster onset of migration was observed on day 1, but not on days 3 and 7. Analyzing migratory behavior in absolute numbers, exposure to RJ EVs released from a collagen scaffold lead to approximately 88% scratch closure on day 1, 3, and 7. Relative to respective intra-experimental control, the RJ EVs group displayed a 44% higher scratch closure on day 1, but only a 15% increase on day 3, and 18% on day 7.

#### RJ EVs increase contractile behavior of HdnFs

**Figure 6.**
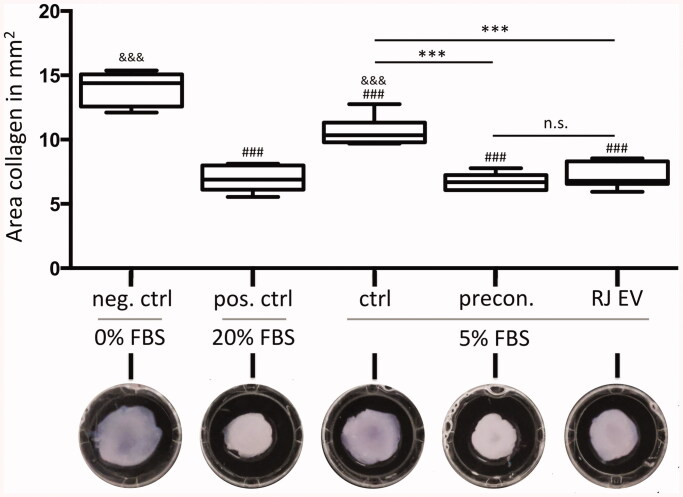
RJ EVs increase contractile behavior of HdnFs. Upper panel: Area of collagen gels containing 1.5 × 10^6^ HdnFs per ml, 24 h after gel polymerization. Negative control (neg. ctrl) was incubated with 0% FBS, positive control (pos. ctrl) in 20% FBS, control (ctrl), RJ EV preconditioned HdnFs (precon.) and RJ EV in collagen gel (RJ EV) in 5% FBS; *n* = 6; * statistical difference to ctrl; # statistical difference to neg. ctrl; and statistical difference to pos. ctrl; mean ± SD statistics described in methods; lower panel representative images of collagen gels after 24 h incubation;.

Interaction of HdnFs in collagen gels with and without RJ EVs was assessed in a collagen contraction assay. As seen in Figure 6, concentration of FBS has a significant effect on the contraction of control gels, resulting in the most pronounced concentration of the positive control 20% FBS (7.0 ± 1.0 mm^2^) and least contraction of the negative control 0% FBS (14.0 ± 1.3 mm^2^). Five percent of FBS group was significantly different to both control groups (10.6 ± 1.1 mm^2^). Both RJ EV groups (RJ EV collagen and RJ EV preconditioned) displayed significantly stronger contraction than the respective control group (ctrl 5% FBS; 7.2 ± 1.0 mm^2^; 6.7 ± 0.6 mm^2^).

#### Biofilm inhibiting effect of RJ EVs released from collagen gels

**Figure 7.**
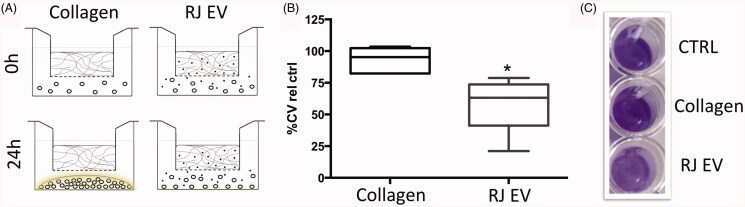
RJ EVs released from collagen gel inhibit *S. aureus* biofilm formation. (A) Schematic representation of experimental setup. (B) Numeric results for crystal violet biofilm staining, expressed as percentage of positive control, for collagen gels with and without RJ EVs (*n* = 4, mean + SD; statistics described in methods. (C) Representative image of crystal violet *S. aureus* biofilm staining.

Inhibition of biofilm formation in *S. aureus* ATCC 29213 is one of the known RJ EV characteristics demonstrated in an earlier study (Schuh et al., 2019). However, the effects have not been assessed in a continuous release system. As seen in Figure 7(B,C), collagen had no significant effect on bacterial growth: collagen group displayed 96.3% of biofilm growth compared to the control group. RJ EVs released from collagen gels however, significantly decreased *S. aureus* biofilm formation (66.9% of control).

## Discussion

Exosome and EV research has gained considerable attention over the last decades. However, although EVs themselves have been studied as delivery systems for drugs or compounds (Ha et al., 2016; Bunggulawa et al., 2018), their targeted delivery using scaffolds has been widely understudied so far.

Recently, our group has demonstrated that RJ EVs display a number of favorable characteristics for wound healing, including antibacterial and biofilm-inhibiting properties in *S. aureus* strains as well as promoting migration in human mesenchymal stem cells (Schuh et al., 2019). The logical next step included the development of a delivery system, since especially in wound healing, topical release systems are a common way of drug delivery (Saghazadeh et al., 2018). Type I collagen, as one of the most commonly used biomaterials (Copes et al., 2019), offers a number of properties favorable to EV release including porosity, fibrous mesh-like structure and high biocompatibility (Parenteau-Bareil et al., 2010). Studies assessing pore sizes of type I collagen report differing numbers, ranging between 1 and 10 µm (Wolf et al., 2013; Fraley et al., 2015). Apparently, pore sizes can vary as a result of the method used for analysis, collagen source, gelling temperature as well as protein concentration, with the latter having the most pronounced influence.

To assess RJ EV release, three low-concentration collagen formulations were tested in regard to their vesicle-releasing capacities for a time course of 7 d. As a first step, EVs isolated from royal jelly were characterized regarding homogeneity in particle size in order to ensure controlled and comparable release from collagen gels. EV batches were selected in order to have minimal differences in size distribution, with median particle sizes between 110 and 135 nm (Figure 1(C)). Several studies report that acidic pH does not compromise EV integrity (Ban et al., 2015; Zhao et al., 2017). However, a later study by Cheng et al. reported that acidic pH around four resulted in a decrease in exosome number and lower detection of exosome associated markers, suggesting a destructive effect on EVs (Cheng et al., 2019). According to manufacturer’s data sheet, type I collagen in acidic buffer displays a pH of around 3.5. Ideal collagen gelling temperature has been reported to be around 37 °C, but occurs at room temperature as well (Achilli & Mantovani, 2010). To avoid compromising structural integrity of the EVs integrated into the matrix, or the collagen gel formation, collagen was neutralized on ice and ice-cold EVs were added subsequently, with polymerization occurring at 37 °C.

Analysis of the released particles revealed no change in particle size compared to the NTA results observed pre-collagen integration (Figure 2(D)), suggesting that the process of integration into neutralized collagen gels does not change EV basic properties. Release dynamics revealed an interesting picture: even though pore size should allow sufficient EVs release, 3 mg/ml collagen gels displayed a cumulative release of only around 3% of EVs, or around 4 × 10^6^ EVs per 100 µl collagen gel (Figure 2(B)). Regarding functionality of RJ EVs, our previous study has shown that in a controlled environment a ratio of 0.1:1 EVs:CFU was not sufficient to exert a biofilm-inhibiting effect (Schuh et al., 2019). Assuming stable release kinetics, EVs are released from 3 mg/ml collagen gels at a ratio of around 3 × 10^4^ per hour, which cannot be expected to efficiently reduce growth of a developing biofilm.

Interestingly, 1 mg/ml displayed a drastic increase of released particles after 3 d, but consistently throughout the independent experiments no further release after 7 d. We hypothesize that collagen concentration impacts EV availability for release: collagen at a concentration of 1 mg/ml displays an initial higher release due to the known larger mesh size, which leads to faster depletion of EVs available for release within the gel. For wound healing applications, a stable release is favorable, hence further experiments were performed with 2 mg/ml collagen gels (Figure 2(A)).

Further characterization of the 2 mg/ml collagen gels with RJ EVs in comparison to the control was done using AFM, a well-known method for nano-characterization of collagen-based biomaterials (Bozec & Horton, 2005; Aguayo et al., 2016; Stylianou, 2017). In the field of EVs, AFM is relatively new, but has significantly broadened the scope of characterization possibilities, i.e. adding label-free-single vesicle morphology as well as insights into quantitative single vesicle adhesivity, elasticity or deformability (Sharma et al., 2018). Interestingly, the interaction between biomaterials and EVs has not been assessed with AFM yet. To facilitate observation of EVs on the surface of the collagen, a mild fixation was chosen, as it is not expected to significantly modify the topographical characteristics of the gel. Visible EVs displayed a spherical shape and appeared to be intact (Figure 3). They were flattened on the top, which can be associated to the nature of the technique, previously shown by Sharma et al. for amplitude images (Sharma et al., 2011). Interestingly, comparing overall topography of the collagen gel with and without EVs, it can be seen that presence of EVs changes collagen gel appearance. Fibers appear bulkier and rounder compared to the control gels, which could be due to EVs entrapped underneath the fibers or due to structural changes within the collagen. Analyzing fiber stiffness, there was no significant difference, however, a trend toward lower fiber stiffness was detected (Supporting Information Figure 1). Further studies are needed to fully characterize potential changes in collagen formation and composition, nevertheless, we report here for the first time a snapshot of EV release from collagen gel matrices using AFM.

Stable release and structural integrity are the prerequisite for EVs utilized in a functional experimental setting. Several studies have analyzed the effects of temperature on EV stability and functionality over time. In an early study in 2011, Sokolova et al. described a decrease in size for EVs stored at 37 °C, starting at day 1 (Sokolova et al., 2011). It was observed that size of released RJ EVs underwent only minor changes over time at 37 °C, allowing to imply a sustained stability and functionality. Therefore, as a next step and proof-of-concept experiment, collagen gels were co-located with a fibroblast monolayer culture to assess uptake into cells (Figure 4). Qualitative analysis confirmed uptake into fibroblasts over the course of one week.

The effect on fibroblast behavior after integration was further assessed in an *in-vitro* wound healing assay, also known as fibroblast migration assay or scratch assay. Fibroblasts were pre-conditioned with RJ EVs released from collagen gels in Transwells, and subsequently their ability to migrate into an inflicted scratch was explored (Figure 5). To exclude migration results being influenced by increased proliferation, a BrdU proliferation assay was performed, verifying indeed a decreased proliferation in presence on RJ EVs. Interestingly, a trend toward faster onset of migration was observed on day 1, leading to almost 40% scratch closure after 12 h. This trend was not observed on day 3 or day 7. Given the increased release rate on day 3 and day 7 with no change in RJ EV size, a more pronounced effect on cellular migration was expected. Nevertheless, after 24 h the RJ EV group displayed around 88% gap closure, on day 1, 3, and 7, which was consistently higher than the respective controls (Figure 5(A)). We hypothesize that on the one hand, as described in various studies, a decrease in EV efficacy over time is probable, especially during incubation at 37 °C. However, it also indicates that RJ EVs maintain a certain activity over the time course of 7 d. On the other hand, interaction between fibroblasts and RJ EVs is not fully understood yet. It is notable that independent of RJ EVs released, results after did not differ significantly (or displayed a trend), but remained around 88% gap closure, so that a self-regulating effect in a dynamic EV release model cannot be excluded (Figure 5(A)). The same effect was seen regarding HdnF proliferation, showing now significant differences between day 1, 3, and 7. Potential effects and mechanisms of RJ EVs on a molecular level, however, remain to be assessed in further studies.

One of the main complications in wound healing is bacterial biofilm formation (Metcalf & Bowler, 2013), especially by clinically relevant strains such as *S. aureus* (Serra et al., 2015). As it is difficult to disrupt fully established biofilms, current efforts are focused on developing new strategies to inhibit or delay the formation of biofilms on surfaces. Thus, we tested the potential inhibitory effect of RJ EVs on biofilm formation in a dynamic EV release system using EV-loaded collagen gels in Transwells, collocated with a standardized *S. aureus* inoculum. We analyzed the possibility of using a Kirby-Bauer-like setup given its highly standardizable nature, however, due to the spherical nature of EVs, it could not be assumed to display the same favorable leach-out characteristics as for molecular compounds in aqueous solution (Cai et al., 2010). The interesting aspect of the Transwell-leach-out system is the undisturbed bacterial growth, analyzing the effect of the released EVs in an isolated manner, as opposed to being in direct contact with the bacteria. It was found that collagen alone had no significant effect on bacterial growth, which is in accordance with other studies analyzing the antibacterial properties of collagen (Michalska-Sionkowska et al., 2017; Ge et al., 2018). Collagen gels containing RJ EVs significantly reduced biofilm formation compared to the control group. Comparing the results to our previously published study, a reduction of biofilm formation by around 45% is within a ratio of 1:1 and 0.1:1 EVs per CFU (Schuh et al., 2019). Given a release of 5.10^6^ EVs within 24 h and assuming a stable release rate of 2–2.5 × 10^5^ RJ EVs per hour for the 2 × 10^5^ CFU bacterial broth, these results are in accordance with our previous findings, and confirm biofilm-inhibiting effects of RJ EVs released from a collagen scaffold.

Assays performed with Transwells provided insight into the functionality of RJ EVs released from collagen gels but are not able to assess cellular interactions with collagen. Collagen contraction assays are a standard method allowing assessment of such interaction (Bell et al., 1979). To verify whether potential effects in the contraction of collagen in presence of HdnF are associated to an altered collagen structure or altered cell behavior, RJ EV preconditioned HdnFs were compared to HdnFs exposed to RJ EVs inside the collagen gel. It was found that both groups displayed a significantly higher ability to contract collagen gels compared to control gels without RJ EVs, independent of preconditioning or availability in the gel (Figure 6). This increased ability of HdnFs to remodel to remodel and contract a collagen matrix promoted by the internalization of RJ EVs could be of interest in the field chronic wounds. Biofilm formation as well as decreased vascular supply are common issues affecting chronic wounds, and known to be the main drivers of delayed wound contraction and closure (Guo & Dipietro, 2010). In this study, we could demonstrate that RJ EVs encapsulated in type I collagen gels are not only accessible to HdnFs but are also significantly increasing their contractibility, while displaying strong antibacterial properties.

In summary, in this study we could demonstrate the suitability of type I collagen as a matrix for sustained and predictable RJ EV release, a crucial step toward developing local delivery systems for EVs in clinical settings. Most importantly, integration into collagen did not alter EV size or integrity, resulting in functional EVs released over the time course of up to 7 d. The combination of RJ EVs and type I collagen has demonstrated highly promising results, which now have to be confirmed in preclinical studies.

## Supplementary Material

Supplemental Material
