# Supplementary figures and images for: Type I collagen hydrogels as a delivery matrix for royal jelly derived extracellular vesicles
Source: Drug Deliv. 2020 Sep 14;27(1):1308–18. doi: 10.1080/10717544.2020.1818880 (PMC7534280; doi:10.1080/10717544.2020.1818880)

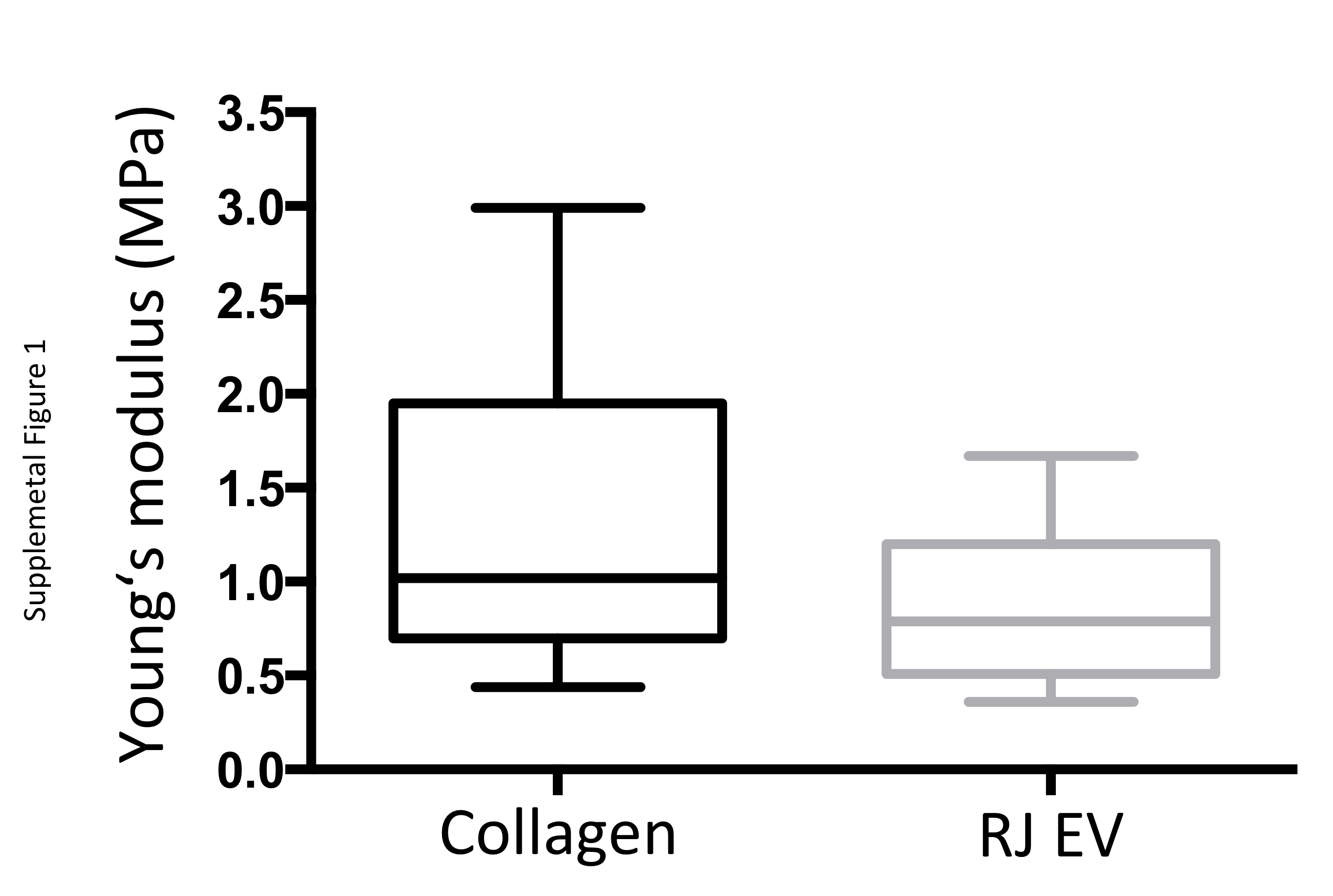

Supplement: Supplemental Material [file IDRD_A_1818880_SM4346.tiff]
